# Treatment Response in Advanced and Metastatic Esophageal Cancer Patients on Palliative Treatments in Tikur Anbesa Specialized Hospital, Addis Ababa, Ethiopia: A Retrospective Study

**DOI:** 10.21203/rs.3.rs-9080266/v1

**Published:** 2026-05-20

**Authors:** Damena Teshome, Adugna Fekadu, Sonia Worku, Hiwot seboksa, Daniel Tesfaye, Wondimagegn Tigeneh, KaleEgziabher Lukas

**Affiliations:** 1 Clinical Oncology Resident, Addis Ababa University, Addis Ababa, Ethiopia; 2 Department of Surgery, Addis Ababa University, Addis Ababa, Ethiopia; 3 Department of Internal Medicine, Addis Ababa University, Ethiopia; 4 Network for Oncology Researches in Africa/NORA initiative, Addis Ababa University School of Public Health

**Keywords:** Treatment response, Oesophageal Cancer, Palliative treatment, Addis Ababa, Ethiopia

## Abstract

**Background:**

Greater than 90 % of oesophageal cancer patients present at an advanced and or metastatic stage in low-income countries including Ethiopia which are also hot spot for this cancer. But there is limited study done on palliative treatment response in Ethiopia. So this study seeks on palliative treatment responses in advanced and metastatic oesophageal cancer patients in the oncology department at TASH.

**Method:**

The study is a retrospective cohort of 70 patients with advanced and metastatic oesophageal cancer patients who took palliative treatment at Tikur Anbesa Hospital from June 20, 2019 to November 10, 2022. Data were retrieved from the Hospital-based Cancer Registry to identify patients with esophageal cancers. The overall survival rate was calculated by the Kaplan-Meir method and the correlates between clinical parameters and prognostic significance were estimated by the long-rank test using SPSS version 26.0. We applied a p-value of 0.25 from bivariate to multivariate cox regression analysis and decided a significant at p value of less than 0.05 at multivariate cox-regression analysis.

**Results:**

Among the total 70 patients reviewed for this study, 97.6% of them had planned treatment and among those 68.8% took chemotherapy, and 28.6% took surgery. The median overall survival was 17 months. Chemotherapy treatment with chemotherapy has shown overall response rate (ORR) of 21.1%, 18.8% and 3.5% at mid-cycle, end-cycle, and 4 months after completion of chemotherapy respectively. The study also showed that there was at least one-grade improvement of dysphagia in 30% at end-cycle of chemotherapy, and 15% of patients after one month of surgery respectively. The study also highlighted treatment without surgery has a 61.6 % decreased risk of esophageal cancer death (AHR=0.384, 95 CI: 0.161–0.913, P<0.03).

**Conclusion:**

Although median overall survival seems higher compared to studies in another part of the world including Africa, Overall response rate and symptomatic dysphagia improvement is very low after completion of chemotherapy and palliative surgery.

## Background

Cancer is the second leading causes of morbidity and mortality worldwide(“Global, regional, and national life expectancy, all-cause mortality, and cause-specific mortality for 249 causes of death, 1980–2015: a systematic analysis for the Global Burden of Disease Study 2015,” 2016)^i^. Oesophageal cancer is the eighth in incidence and the sixth in mortality in the world (Sung et al., 2021). Although, the disease burden varies across countries, it is one of the cancers with high morbidity and mortality across the world. About 604,100 new cases and 544,100 deaths estimated in the same year worldwide. Global variation in incidence and mortality observed the highest south eastern Asia, south and east Africa and lowest in West Africa and Central America. If rates remain stable, 957,000 new cases and 880,000 deaths are expected from oesophageal cancer in 2040(Cheng et al., 2015; Morgan et al., 2022). A cross-sectional study found that a total of 919 patients in Tikur Anbesa Hospital from 2010 to 2014 with oesophageal cancer and it is the third most common cancer in males (Solomon & Mulugeta, 2019).

Mostly advanced oesophageal cancer patient present complaints of dysphagia to some degree (90%), weight loss and odynophagia (30 to 60 %) and other nonspecific symptoms and signs(Bulcha et al., 2018). Combined with poor set-up for diagnosis in low-income countries most of cases are advanced disease. Which are unresectable/in-operable rather are candidates for palliative treatment. More than 50 % of oesophageal cancer patients have inoperable disease at diagnosis (Lai et al., 2018). This figure is expected to be higher in poor set-up areas.

Management of patients with metastasis and locally advanced disease who are not fit for chemoradiotherapy is mainly palliative. The survival of this group of patients is minimal so treatment goals are symptomatic relief, decreasing disease burden, improving quality of life and prolonging survival. There are many options of treatments developed globally each with its merit and disadvantages. Endoscopic balloon dilatation, laser ablation, Photodynamic therapy, rigid stenting, Self expandable metalic stent, Intraluminal branchy therapy, External beam radiotherapy, bypass surgery, percutaneous feeding gastrostomy/jejunostomy, systemic chemotherapy, targeted therapy and immunotherapy are the options developed to deal for these group of patients so far.

Patients with metastatic stage IV disease and non-metastatic oesophageal cancer categorized for palliative treatments are candidates for further ancillary tests. Unfortunately, health setups in these high-risk areas are not well equipped with treatment options available to deliver the palliative treatment the patient needs, particularly in Ethiopia.

Knowledge of treatment response for each palliative treatment modality with its limitation is important to individualize the treatment. There are many researches done showing the response of patients for different modality of palliative treatment option mostly in advanced set up(Homs, Kuipers, & Siersema, 2005). But in poor set up like Ethiopia the available treatment option are mostly chemotherapy ,external beam radiotherapy and surgery(Hassen, Teka, & Addisse, 2021). Rarely, stenting is available in few private centers with relatively expensive cost for majority of the patients. Besides, the most common palliative treatment in our set up chemotherapy is low dose regimen compared to the standard globally applied dose because of poor performance and frail nature of our patient with malnutrition which guarantee further response evaluation. The output of this research warrants a change in clinical practice with consideration of alternative treatment. Further, it can be an eye opener for further prospective and quality of life based studies in our country. Finally, local standard guideline could be developed.

## METHODS AND MATERIALS

### Study area

The study was conducted in Tikur Anbessa Specialized Hospital (TASH), which is a government owned tertiary referral and teaching hospital located in the Ethiopian capital, Addis Ababa.It is the only hospital in the country which provides comprehensive cancer care,surgery,RT,chemotherapy and palliative service at the start of this study. Oncology department is among one of the major departments with high burden of patients in the hospital. The department provides inpatient, outpatient, emergency and palliative care services in the hospital. The oncologic unit is equipped with 2 cobalt machines (1 brachytherapy and 1 EBRT) and 1 functional linac machine.Additionally,there are 31 beds in this department for chemotherapy service and emergency resuscitation. The average numbers of eosophageal cancer patients before the starting of this study was 110 and among them 82 were advanced cases based from hospital’s patient log book list. The study was conducted from January 1, 2022 to November 30, 2022 G.C.

### Study design

Institutional based retrospective study was conducted to assess palliative treatment types delivered and patient outcomes at Tikur Anbesa specialized hospital.

### Source population

All advanced and metastatic oesophageal cancer patients seen in Tikur Anbesa specialized hospital in the study period.

### Study population

All advanced/metastatic oesophageal cancer patients who started palliative treatments at Tikur Anbesa Specialized hospital, from June 20, 2019 to November 10, 2022.

### Inclusion and Exclusion criteria

All advanced and metastatic oesophageal cancer patients who visited TASH in the study period were recruited.The inclusion criteria included all biopsy confirmed eosophageal cancer patients, who took 1 or more mode of palliative treatment options in TASH.Those patients with locally advanced stage and received curative intent of treatment and patients with poor performance who were on supportive care were excluded from the study. Those patients with imaging diagnosis of oesophageal cancer but without biopsy were also excluded from the study.The other exclusion criteria were incomplete medical records(unknown date of biopsy,missing sociodemographic feutures like age,sex;clinicopathologic characteristics like location of tumor,performance and grade of dysphagia).Those patients with no record of follow up after palliative treatments were also excluded.Patients who did not give verbal consent were not also part of the study.

### Sampling techniques and sample size

All patients with advanced/or metastatic oesophageal cancer who took at least 1 mode of palliative therapy and fulfilling the inclusion criteria from June 20, 2019 to November 10, 2022 were included. Out of 284 patients with advanced and metastatic oesophageal cancer patients identified in the study period about 115 were excluded because patients were on supportive care only.The other 99 patients were excluded because of missing biopsy and biopsy date,incomplete medical records and refusal of consent. Only 70 patients were identified who fulfilled the inclusion criteria and entered in to the study for analysis.

### Data collection instrument

Data was collected using a structured questionnaire which was adopted from literature with some modification to ensure applicability to our current study in English language. Pre-test was done on 5 patients in oncology department at TASH and those participants chart were excluded during actual study. Some modifications were made on questionnaires based on the pre test result.The questionnaire consists of questions on socio-demographic characteristic, clinical factors, mode of treatment, response after 1 and 4 month for surgically treated patients, after mid and end cycle response for chemotherapy treated patients, change in clinical findings and survival status.

### Data collection method

HMIS logbooks and charts were reviewed to identify patients with advanced and metastatic oesophageal cancer patient who took palliative treatments in oncology department in the study period. Two resident physician were trained on the purpose and how to extract data from patient chart and phone interview. Data was collected by trained residents on the data collection tools. After reviewing charts that fulfilled the inclusion criteria, phone call was made and verbal consent was taken. The outcome variables such as change in dysphagia, performance, clinical and imaging response after each and specified period ,and independent variables were extracted. Date of death was enquired and survival calculated from date of biopsy diagnosis up to the occurrence of events.

### Variables in the study

#### Dependent variables:

(Death, performance and grade of dysphagia after treatment,objective response and clinical benefit)

#### Independent variable:

Socio-demographic data; Age, sex, marital status, occupation; Clinico-pathologic data; Performance before treatments, histologic type, location of tumor, stage of disease; and Co morbid illness: Treatment modality, type of chemotherapy, time to treatment initiation

### Data quality control

The questionnaires were reviewed for completeness on daily basis by the supervisor. Medical records with incomplete patient data were excluded from the study. Further, the questionnaires had been pre-tested on 5% of sample in similar setting, to ensure a reliability of the data collection tools.

### Data analysis

After data completeness was checked, we entered data into kobo tool box ,then imported into SPSS version 26 and analysis done. Descriptive summary of the data presented in frequency tables, figures and graphs. Continuous variables also reported as means and standard deviations. Chisquare tests were made to identify relationship between outcome variables ,that is,survival and predictor variables.We applied Kaplan meir to identify survival of patients over time and log rank test done to test its significance.Cox regression analysis applied to identify predictors variables for outcome variable.Those variables which had an association with p value< 0.25by univariate analysis were entered into multivariate and P< 0.05 was considered statistically significant to establish the association between treatment outcome and predictor variables.Results were expressed using HR and 95% CI with p value < 0.05.

### Ethical consideration

Ethical clearance was sought from institutional ethical review board of school of public health of Addis Ababa University. Clearance letter (AAU/Onc/12/2022) was given from oncology department, TASH, Addis Ababa University before proceeding with data collection. Patients’ information had been kept confidential and solely used for this research. Names of patients were not included in the questionnaire rather code numbers were used. No one except the members of the research team has an access to the collected information. All papers of the study have been kept in a secured place under lock and computer records were locked with passwords throughout the course of this study.

## Result

### Socio-demographic characteristic

Among the total 70 participants, 35(50%) were male and the mean age of the participants was 51.40 years SD ±11.513. From the total participants, 60 (85.7%) of the study participant were married and about 50 (71.5%) were came from Addis Ababa. Only 12 (17.1%) of the study participant had common habit of drinking alcohol, smoking cigarettes or both. In addition to esophageal cancer about (11.4%) of participant has associated chronic comorbid illness. Participant has different occupation and roles (38.6%) housewife, 16 (22.9%) farmer, 12 (17.1%) government employee, and 15(21.4%) were merchant. (Table 1)

### Clinico-pathologic characteristics of participants

Histologically 69 (87.1%) patients were diagnosed with eosophageal squamos cell carcinoma (SCC) and the remaining adeno carcinoma. At diagnosis about 59 (85.5%) were stage 4 and 9(13%) were stage 3. 5(7.1%) cervical 9(12.9%) has upper thoracic, 25(35.7%) mid thoracic, and 31(44.3%) had distal thoracic/GEJ cancer. On physical examination only 9(12.9%) had pertinent finding on cervical/supra clavicular, chest or abdominal finding. The most common form of presentation was dysphagia in 69(98.6%) of study participants. (Table 2)

### Palliative treatment types patients took

From 70 participants in the study, 20(28%) of them underwent palliative surgery and 68 (97%) of the participant took chemotherapy as palliative treatment. None of the participant in the study took other form of palliative treatment like stenting or radiotherapy. Among surgery group 18 underwent feeding gastrostomy or jejunostomy and the remaining 2 had palliative resection and anastomosis. There was difference in time from diagnosis to palliative surgery with a mean of 1.21 ± 0.976 months, range (0 to 4 months). The majority of participant 68(97.1%) (N=70) has taken some form of chemotherapy. About 39 (57.4%) took cisplatin/pacliataxol, (16) (23.5%) took cisplatin/5-fu, 8(11.8%) took carboplatin/paclitaxol, 4(5.9%) took folfox, and one patient (1)(1.5%) took other form of chemotherapy. The average interval between diagnosis and initiation of chemotherapy was 3.1 months with (SD±2.045 months), ranging from 1 to 13 months. (Table 3)

### 5.4 Subjective and objective treatment responses

**Figure 2:**
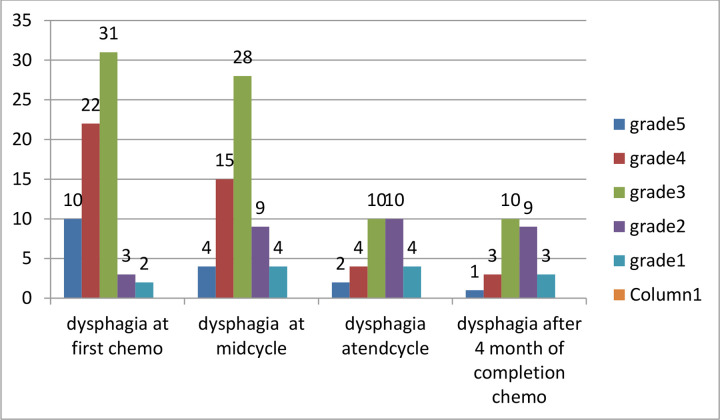
Grade of dysphagia at first cycle chemotherapy,at mid cycle,end cycle and after 4 month of completion chemotherapy in advanced and metastatic esophageal cancer patient who took chemotherapy from June 20,2019 to November 10,2022,in TASH, Addis Ababa, Ethiopia.

**Figure 3:**
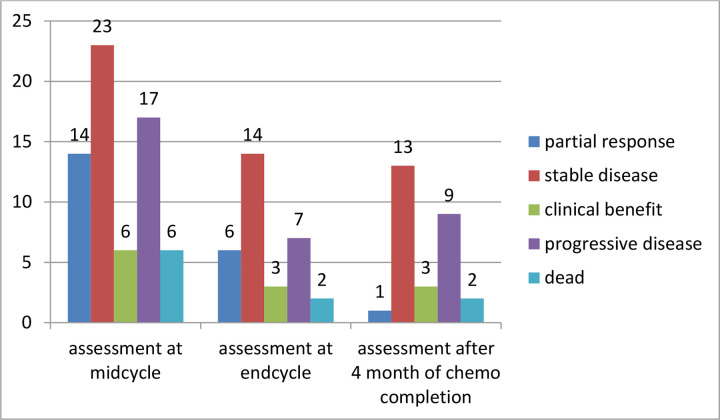
Assessment of disease at mid cycle, end cycle and 4 month completion of chemotherapy in advanced and metastatic esophageal cancer patient who took chemotherapy in TASH, Addis Ababa, Ethiopia from June 20,2019 to November 10,2022.

From 20 patients in the study who underwent surgery, 3(15 %) had at least one grade of dysphagia improvement after 1 month of surgery, but there was no change in grade of dysphagia after 4 months compared to after 1 month of surgery. Also, 9 (45%) from those who underwent surgery has improvement in performance status from ECOG -II to ECOG -I ,after 1 month of surgery but no change after 4 months compared to 1 month after surgery. From all twenty (20) who had surgery only 2 (10 %) has clinical benefit after 1 and 4 month of surgery, while 2(10%) had progressive disease after 4 month of surgery. (Figure 2 and figure 3)

**Figure 4:**
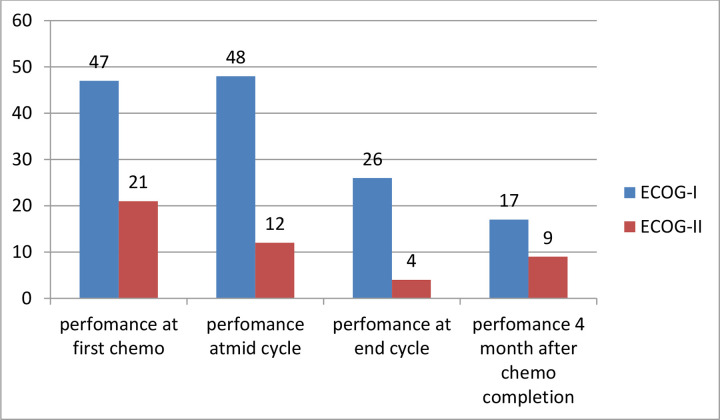
Performance status at first chemotherapy, at mid cycle, at end cycle and after 4 month of chemotherapy completion in advanced and metastatic esophageal cancer patient treated in TASH, Addis Ababa, Ethiopia from June 20, 2019 to November 10, 2022

Patients who took chemotherapy had dysphagia of grade IV and above in 19 out of (n=60) patients at mid cycle of chemotherapy compared to thirty two (32) (47%) out of (n=68) before chemotherapy. There was at least one grade of dysphagia improvement in 15 (25%) (n=60) patients at midcycle of chemotherapy compared to at first cycle of chemotherapy. There was grade IV and above dysphagia in 9(30%) out of 30 evaluable patients at end cycle of chemotherapy. After 4 month of chemotherapy only 4 patients had grade IV and above dysphagia from 26 evaluable patients. (figure 4

Palliative chemotherapy yielded different response at different assessment period. At mid cycle, twenty-three (23), fourteen (14) and six (6) out of sixty six (66) evaluable patients had stable disease, partial response and clinical benefit, respectively. In addition, seventeen (17) and six (6) out of same sixty six (66) patients had progressive disease and dead at mid cycle assessment, respectively. At end cycle assessment fourteen (14), six (6), and three (3) out of 32 evaluable patient had stable disease, partial response and clinical benefit, respectively. With the same assessment time 7 and 2 out of (n= 32) had progressive disease and dead, respectively. After 4 month of end of chemotherapy from evaluable (n=28) patients 13, 1 and 2 had stable disease, partial response and clinical benefit respectively, while 9 and 2 patients had progressive disease and dead, respectively. Hence ORR for palliative chemotherapy in this study is 21.1%, 18.8%, and 3.5 % at mid cycle, end cycle and after 4 month of completion chemotherapy respectively. (Figure 5)

**Figure 6:**
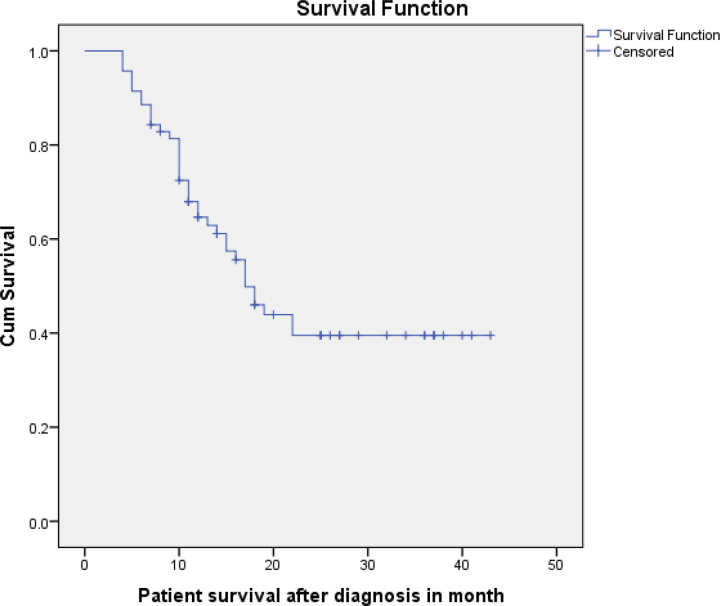
Kaplan meier overall survival curve in months for advanced and metastatic esophageal cancer patients taking palliative treatment in oncology department at TASH, Addis Ababa, Ethiopia from June 20,2019 to November 10, 2022.

Performance status change was noted at different assessment period of chemotherapy treatment. All patients who took chemotherapy had performances status of ECOG- I and ECOG-II. At mid cycle assessment twelve (12) (20%) out of sixty (n=60) patients had ECOG-II compared to twenty one (21)(30.9%) out of sixty eight (n=68) patients at start of chemotherapy. End cycle assessment showed four (4) (14.8%) out of (n=30) had ECOG-II performance status. While after 4 month of chemotherapy completion 9 out of (n=26) patients had ECOG-II performance status. (Figure 6)

### Overall survival

From the total patients involved in the study, the prevalence of mortality rate was 52.9%, 95 % CI (41%, 65%) with median survival of 17 months 95%CI (13.75, 20.24) months. This means among 100 advanced and metastatic oesophageal Cancer patients on palliative treatment in oncology department at TASH, almost 53 of them died: and almost 50% of the total patients survived until 17 month since there diagnosis date. The overall survival curve as below (Fig 6).

### Factors associated with mortality in advanced and metastatic esophageal cancer taking palliative treatments

The rate of survival was different along categories of covariates such as occupation, endoscopic tumor location, initial treatment plan, treatment with surgery, no chemotherapy, time to chemotherapy initiation and completion of full cycle chemotherapy. But significant association was found with endoscopic tumor location, initial treatment plan and treatment with surgery. The survival varied with tumor location, cervical esophageal cancer having worst median OS of 10 months (95%: CI (5.7, 14.3), log rank test, p=0.048). Survival also varied with initial treatment plan assignment those planned for Surgery and RT showing median OS of 10 months (95%: CI (7.19, 12.81),log rank test, p=0.001). Patients survival also varied with surgery intervention, median OS of 12 months (95%: CI (9.8, 18.18), log rank test, p=0.001).

However, in multivariate cox proportional hazard model only treatment with no surgery determined esophageal cancer survival independently. Treatment with no surgery showed 61.6 % decreased risk of esophageal cancer death than those who underwent surgery (AHR=0.384, 95% CI: 0.161–0.913, P<0.03).

### Bivariable and Multi-variable Cox-regression analysis

Both bivariate and multivariable cox-regression analysis was performed to identify factors associated with overall survival for advanced level esophageal cancer patients. In bivariate cox regression analysis, Patient underwent surgery, Co morbidity, Endoscopy location, and treatment plan were fitted for multivariable cox-regression analysis with the p value of < 0.25. However, in multivariate cox proportional hazard model only treatment with no surgery determined esophageal cancer survival independently. Treatment with no surgery showed 61.6 % decreased risk of esophageal cancer death than those who underwent surgery (AHR=0.384,95% CI:0.161–0.913, P<0.03).

## DISCUSSION

This study highlighted clinicopathologic feature, palliative treatment types, and subjective and objective response for each palliative therapy type in advanced and metastatic esophageal cancer patients treated in Tikur Anbesa hospital. This study discerned that the dominant histology was sc 61(87.1%), dysphagia is the commonest presentation in 69(98.6%), mid and distal/GEJ location is commonest site in 56(80%) and commonest area patients are coming from Oromia region in 41(58.6%) of patients. This finding is in line with study done in Arsi zone, Oromia region in 2021(Deybasso, Roba, Nega, & Belachew, 2021).The finding is also in line with previous study in Tikur Anbesa hospital (Hassen et al., 2021).This could be due to hypothesized hot cultural diet in those hot spot area SCC being the most common histology.

Most of the patients in the study took chemotherapy alone as palliative treatment for dysphagia. There was 25% decrement of grade iv and above dysphagia at midcycle compared to at chemotherapy initiation. The overall response rate however was small 21.1 % at mid cycle, 18.8 % at end cycle and 3.5%after 4 month of chemotherapy completion. Clinical benefit observed was 9% at mid cycle and end cycle of chemo and 11% after 4 month completion of chemo. This response is small compared to study done in Germany with over all response rate of 40% and clinical benefit of 70% with cisplatin/paclitaxol regimen (Petrasch et al., 1998).Though most of the participants in our study took cisplatin/pacliataxol the dosage and intensity is different to the one done in Germany. The low response rate for chemotherapy in the study compared to other study may be due to moderately low dose of chemotherapy due to patient presenting with late stage with poor nutritional condition and fear of toxicity and set up limitation(lack of infuser for 5-fu).

Chemotherapy showed at least grade 1or above dysphagia improvement in 30 % of patients after completion of chemotherapy and palliative surgery showed grade 1or above improvement in 15 % after 1 month of surgery. This is small response for palliative dysphagia compared to study in Sweden in which dysphagia response was 49% in chemotherapy,56% in radiotherapy and 81% of stent insertion(Cwikiel, Cwikiel, & Albertsson, 1996).Dysphagia response is also lower compared to short course accelerated radiotherapy(SHARON II) study done here in Tikur Anbesa hospital in which 76 % partial or complete improvement for dysphagia was seen after 1 month of completion radiotherapy with no>_G3 toxicity(Deressa et al., 2020).This tells that palliative local treatments with stenting or RT could offer more local symptom(dysphagia) relief than systemic therapy.

The median overall survival of patients taking palliative treatment for advanced and metastatic esophageal cancer patient was 17 months. This finding is closer to the one reported in China ^ii^in which patient treated by CCRT vs. chemotherapy alone in metastatic esophageal cancer patient median OS was(12.9 vs. 9.3 months) respectively (Li et al., 2022). Optimal management of esophageal cancer in Africa; A systematic review also reported survival of advanced metastatic esophageal cancer patient range from 4 to 41 weeks (Buckle et al., 2021). This is far from our study finding. However, our finding is much higher than one study done here in Tikur Anbessa hospital reported median OS of 4 months all stages of esophageal cancer combined(Hassen et al., 2021). As general the OS(median)is higher than reported for advanced stage/metastatic esophageal cancer elsewere. This may be due to lack of inclusion of poor performance patient on best supportive care which would have low median OS. Although not analyzed separately most included patient are in operable with no metastasis. Locally advanced in operable esophageal cancer are staged with CT scan showing local infiltration or loss of fat plane between the mass and local structure like airway, great vessels, heart and vertebral body. Most of the patients in our set up are frail ,thin patients with low fat content as general so clear fat plane between structures may not be seen normally(Schneekloth, Terrier, & Fuchs, 1983).As a result, most of locally advanced stage diagnosis without adjacent organ infiltration by mere loss of fat plane could have been up staged(Quint, Glazer, Orringer, & Gross, 1985).Further study is needed to address this well.

Treatment with no surgery is the independent determinant of survival in advanced and metastatic esophageal cancer patients who took palliative treatment in the study. Those with no surgery as treatment has 61.6% of decreased risk of esophageal cancer mortality(AHR=0.384,95% CI:0.161–0.913,P<0.03).The study in USA showed palliative surgery has lower OS compared to non-surgical palliative treatment mode(chemotherapy and radiotherapy) (Coffey et al., 2021).The median OS being low(4month)in previous study in Tikur Anbesa hospital(Hassen et al., 2021) compared to our finding could be due to surgical intervention in which radical surgery done (84%) and feeding tube(31.9%) with advanced stage disease(69% stage IV and 19 % stage III) in the former group. Patients subjected to surgery particularly in our set up are those with high grade dysphagia (grade IV and above) which means high intraluminal or wall thickening and may be high burden of disease than those with low grade dysphagia which do not necessitates surgery even within same stage groups.

### Strength of the study

The strength of the study is that it shows the overall survival rate though limited sample size and the possible factors affecting it in the study area. The study tried to address the objective and subjective responses for limited palliative treatment options available in our set up..

### Limitation of the study

The limitation of these study is it had limited sample size could hinder the power to detect difference between groups. Due to retrospective study design and poor documentation of grade of symptoms ,it requires to be sensitive in interpreting especially symptomatic responses.

## Conclusion

Overall this study describes that treatment with chemotherapy has shown over all response rate (ORR) of 21.1%, 18.8% and 3.5% at mid cycle, at end cycle and 4 month after completion of chemotherapy respectively. The study also shows that there is at least one grade improvement in dysphagia in 30% and 15% of patients at end cycle of chemotherapy and after one month of surgery respectively. The study also highlighted survival is better without surgery. Treatment with no surgery is the independent determinant of survival in advanced and metastatic esophageal cancer patients who took palliative treatment in the study.

## Supplementary Material

Supplementary Files

This is a list of supplementary files associated with this preprint. Click to download.

- supplementaryquestioners.docx

## Figures and Tables

**Figure 1:**
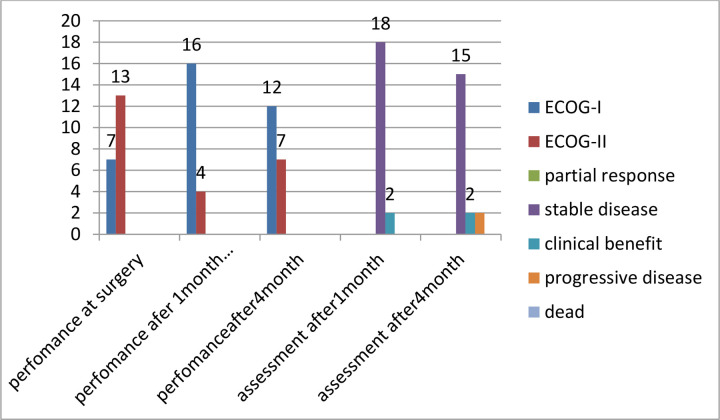
Performance and assessment in those patients who underwent surgery for advanced and metastatic esophageal cancer patient in TASH, Addis Ababa, Ethiopia, from June 20, 2019 to November 10, 2022.

**Figure 7:**
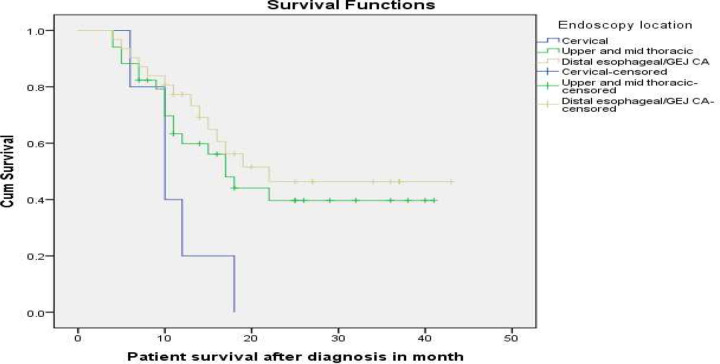
Kaplan meier survival curve based on tumor location for advanced and metastatic esophageal cancer patient taking palliative treatments in oncology department at TASH, Addis Ababa, Ethiopia from June 20,2019 to November 10, 2022.

**Figure 8:**
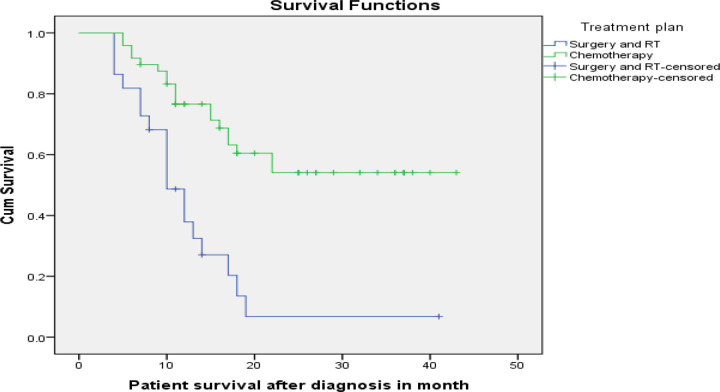
Kaplan meier survival curve based on initial treatment plan for advanced and metastatic esophageal cancer patient taking palliative treatments in oncology department at TASH, Addis Ababa, Ethiopia from June 20, 2019 to November 10, 2022.

**Figure 9:**
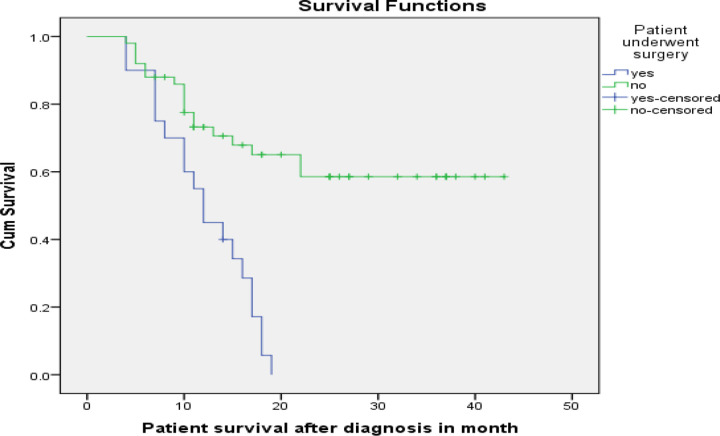
Kaplan meier survival curve treatment with surgery for advanced and metastatic esophageal cancer patient taking palliative treatments in oncology department at TASH, Addis Ababa, Ethiopia from June 20, 2019 to November 10,2022.

**Table 1;:** sociodemographic characteristic of advanced and metastatic esophageal cancer patient taking palliative treatment in TASH, Addis Ababa, Ethiopia, from June 20 to November 10 2022(n=70)

Variables		Frequency	Percentage (%)
**Sex**	Male	35	50.0
	Female	35	50.0
**Region**	Addis Ababa	20	28.5
	Oromia	43	61.5
	Other	7	10
**Marital status**	Married	60	85.7
	Divorced or Widowed	7	10.0
	Single	3	4.3
**Substance use**	No	58	82.9
	Yes	12	17.1
**Co morbid illness**	No	62	88.6
	Has	8	11.4
**Occupation**	Housewife	27	38.6
	Farmer	16	22.9
	Government Employee	12	17.1
	Merchant	15	21.4

*: Amhara, SNNPR, Somale, Tigray, Afar, Benishangul, Gambela, Harari, and DireDawa

**Table 2;:** clinic pathologic variables of advanced and metastatic esophageal cancer patient taking palliative treatment in TASH, Addis Ababa, Ethiopia, from June 20,2019 to November 10,2022 (n=70)

Clinic pathologic variables		Frequency	Percentage (%)
**TNM Staging/imaging**	3	9	13.0
	4	59	85.5
**Histology**	SCC	61	87.1
	Adenocarcinoma	8	11.4
	Poorly differentiated histology	1	1.4
**Endoscopy Location**	Cervical	5	7.1
	upper thoracic	9	12.9
	mid thoracic	25	35.7
	distal thoracic/GEJ	31	44.3
**Clinical Presentation**	Dysphagia	69	98.6
	Weight loss and chest pain	1	1.4
**Pertinent Physical Exam Finding**	cervical/supraclavicular lap	4	5.7
	chest finding	2	2.9
	abdominal finding	3	4.3
	no specific finding	61	87.1
**Performance Status**	1	51	72.9
	2	19	27.1

* Histology is poorly differentiated

**Table 3:** Palliative treatment types planned and delivered for advanced and metastatic esophageal cancer patients in TASH, Addis Ababa, Ethiopia from June 20, 2019 to November 10, 2022.

Treatment type planned and delivered		Frequency	Percentage (%)
**Treatment plan**	Surgery	17	26.6
	RT	3	4.7
	Chemotherapy	44	68.8
**Underwent surgery**	Yes	20	28.6
	No	50	71.4
**Surgery Type**	Feeding gastrostomy or jejunostomy	18	90.0
	Palliative resection and anastomosis	2	10.0
	No	70	100.0
**Take Chemotherapy**	Yes	68	97.1
	No	2	2.9
**Type of Chemotherapy**	Cisplatin/paclitaxel	39	57.4
	Carboplatin/paclitaxel	8	11.8
	Cisplatin/5-fu	16	23.5
	Folfox	4	5.9
	carboplatin/docetaxol	1	1.5

**Table 4:** Grade of dysphagia after 1 month and after 4 months of surgery in advanced and metastatic esophageal cancer patient who underwent surgery from June 20, 2019 to November 10, 2022, in TASH, Addis Ababa, Ethiopia.

Grade of dysphagia after 1 month	Grade 6	7
	Grade 5	7
	Grade 4	3
	Grade 3	3
Grade of dysphagia after 4 months	Grade 6	5
	Grade 5	10
	Grade 4	1
	Grade 3	3

**Table 4:** Bivariate survival analysis using log rank test of advanced and metastatic esophageal cancer patient taking palliative treatments in oncology department at TASH, Addis Ababa, Ethiopia from June 20, 2019 to November 10, 2022.(n=70)

		Censored	Death	Median OS (95%CI)	P value
Underwent surgery	Yes	1	19	12(9.8, 14.18)	0.001**
	**No**	**32**	**18**	30.6(26.2, 35.70)	
Endoscopy location	Cervical	0	5	10.0(5.7, 14.3)	0.048*
	Upper and mid thoracic	16	18	17.0(12.5, 21.5)	
	Distal esophageal/GEJ CA	17	14	22.0(15.8, 29.8)	
Interval between diagnosis to chemotherapy	≤1 month	11	5	26.6(18.3, 34.85)	0.64
	> 1 month	18	30	15.0(11.2, 18.80)	
Treatment plan	Surgery and RT	4	18	10.0(7.19, 12.81)	0.001**
	Chemotherapy	29	19	26.25(21.5, 30.94)	
Occupation group	Merchant	4	11	16.0(13.78, 18.21)	0.077
	Other	29	26	29.3(24.80, 30.89)	
Patient take Chemotherapy	yes	33	35	18.0(12.72, 23.28)	
	no	0	2	6.50(1.06, 18.32)	0.23
Completed Full Cycle Chemo	yes	17	15	22.0(16.72, 28.44)	0.157
	no	13	22	14.0(9.26, 18.74)	

**Table 5:** Bivariate and multivariable cox-regression analysis in palliative esophageal cancer patient treated in Tikur Anbesa hospital from June20, 2019 to November 10, 2022(n=70)

Variables	Crosstab		Unadjusted				Adjusted			
	Censored	Death	HR	95% CI		p-value	HR	95% CI		P-value
**Patient underwent**										
surgery										
Yes	1	19	1				1			
**No**	**32**	**18**	**0.253**	**0.129**	**0.499**	**0.000**	**0.384**	**0.161**	**0.913**	0.03*
**Co morbidity**										
No co morbidity	31	31	1				1			
Has co morbidity	2	6	1.75	0.73	4.22	0.213	1.13	0.46	2.784	0.789
**Endoscopy location**										
Cervical		5	1				1			
Distal thoracic	17	14	0.664	0.34	1.29	0.228	0.65	0.32	1.33	0.241
**Treatment plan**							1			
Surgery and RT	4	18	1							
Chemotherapy	29	19	0.001	0.27	0.14	0.52	0.54	0.22	1.33	0.182

*: Significant at p value of <0.05

## Data Availability

The datasets used and/or analysed during the current study are available from the corresponding author on reasonable request

## Abbreviations

AHR: Adjusted Hazard Rate
EBRT: External Beam Radiation Therapy
SCC: Squamous Celle Carcinoma
RT: Radiotherapy
TASH: Tikur Anbessa Specialized Hospital
